# Editorial: Emerging perspectives in psychiatry: innovations and insights from early career researchers

**DOI:** 10.3389/fpsyt.2026.1909981

**Published:** 2026-07-02

**Authors:** Gaia Sampogna, Bianca Della Rocca, Martina Roijnic Kuzman, Andrea Fiorillo

**Affiliations:** 1 Department of Psychiatry, University of Campania “L. Vanvitelli”, Naples, Italy; 2 Zagreb School of Medicine and Zagreb University Hospital Centre, Zagreb, Croatia

**Keywords:** early career academic, European research, innovation, personalization, shared decision making

Psychiatry is currently moving through a phase characterized by radical changes in clinical presentation, diagnostic formulation and treatment options due to the advances in neuroscience, digital health, psychopharmacology, epidemiology, and public mental health care. At the same time, psychiatry as discipline is increasingly called to respond to complex social, cultural, and health-system challenges, including inequalities in access to care, stigma, the long-term consequences of global economic crises, and the need for more person-centered and collaborative model of treatments (1). Within this evolving framework, early career psychiatrists and mental health researchers have a crucial role to play, with their work often sitting at the intersection between established clinical knowledge and emerging research priorities, bringing new questions and perspectives to the discipline (2).

The Research Topic “Emerging perspectives in psychiatry: research by early career scientists and psychiatrists worldwide” has been conceived as a platform for the young researchers to make contributions in the mental health field. It aims to collect high-quality scientific work across different areas of psychiatry and neuroscience, highlighting the vitality of the new generation of researchers working in collaboration with senior academics.

This Research Topic also underscores the value of mentorship and international collaboration, being hosted under the auspices of the European Psychiatric Association. The Topic Editors are part of the Executive Committee of EPA, being respectively the President, the Treasurer and the Chair of the Early Career Psychiatrists' Committee (ECPC). The commitment of EPA Board members to disseminate research studies with innovative hypothesis and models, tested by solid methodologies as well as to promote educational and training activities, represents one of the main pillars of the new Presidential agenda (3).

By involving early career psychiatrists and scientists from different European countries and different scientific backgrounds and expertise, the present Research Topic can enrich the discipline combining interdisciplinary dialogue, methodological innovation, and diverse perspectives.

Papers included in this volume reflect the complexity of contemporary research in psychiatry, ranging from biological markers and treatment algorithms to service implementation, shared decision-making, stigma, neuropsychiatry, and long-term clinical outcomes.

Several included articles deal with the need for more personalized treatment strategies in psychiatry, such as the systematic review by Rkman et al. on the adoption of structured decision-making for improving treatment outcomes in patients suffering from depressive disorders and the mini-review by Cavaleri et al. analyzing the impact of emerging biomarkers in improving the personalized treatment selection. The paper by Bağcaz & Özel highlights the importance of assessing the impact of any pharmacological treatments in terms of tolerability, adherence, and real-world clinical complexity, not just symptom reduction.

Other papers deal with the impact of physical comorbidities on the long-term quality of life of patients with severe mental disorders (Cipolla et al.), with the need for the development of dedicated interventions for adolescents and young people (Longo et al.) and for implementing evidence-based psychological interventions in public mental health systems (Kince-Laus et al.).

In Europe, since many years the topic of clinical decision making has been studied by the seminal project called CEDAR (4) and in the present Research Topic, the need for improving the shared decision making in clinical practice has been analyzed in the paper by Carbone et al. Further topics include challenging stigma attached to mental disorders (Őri et al.), the description of different suicide risk profile in clinical practice (Cinesi et al.), and the implementation of organizational innovation in psychiatric care in different national contexts (Lacerda et al.).

The articles included in this Research Topic demonstrate that early career researchers are contributing to psychiatry across multiple levels: from molecular biomarkers to clinical phenotyping, from pharmacological treatment to digital psychotherapy, from patient-clinician communication to public mental health implementation. Importantly, the Research Topic shows a bright future for psychiatry, toward integrated knowledge and approaches. Biological innovation will need to be accompanied by attention to lived experience, social determinants, health-system constraints, and cultural context. Similarly, advances in treatment will be most meaningful when they are translated into accessible, acceptable, and person-centred care.

In conclusion, “Emerging Perspectives in Psychiatry: Research by Early Career Scientists and Psychiatrists Worldwide” offers a snapshot of a dynamic and evolving field. The contributions included in this Research Topic show how young researchers are already shaping the future of psychiatric science and practice. By bringing together studies on treatment, precision medicine, implementation, stigma, shared decision-making, neuropsychiatry, and clinical risk, this Research Topic reflects both the complexity of mental health care and the promise of a new generation committed to advance it.
